# Gene Expression Signatures Associated with Pterygium Recurrence

**DOI:** 10.3390/jcm15187257

**Published:** 2026-09-18

**Authors:** Martina Paradzik Simunovic, Marina Degoricija, Jelena Korac-Prlic, Marko Simunovic, Mladen Lesin, Robert Stanic, Livia Puljak, Ivana Olujic, Josipa Marin Lovric, Ana Vucinovic, Zana Ljubic, Kajo Bucan, Janoš Terzić

**Affiliations:** 1 Department of Ophthalmology, University Hospital of Split, Spinciceva 1, 21000 Split, Croatia; martina.paradzik@gmail.com (M.P.S.);; 2 Laboratory for Cancer Research, School of Medicine, University of Split, Soltanska 2, 21000 Split, Croatia; 3 Department of Medical Chemistry and Biochemistry, School of Medicine, University of Split, Soltanska 2, 21000 Split, Croatia; 4 Department of Pediatrics, University Hospital of Split, Spinciceva 1, 21000 Split, Croatia; 5 Department of Pediatrics, School of Medicine, University of Split, Soltanska 2, 21000 Split, Croatia; 6 Center for Evidence-Based Medicine, Catholic University of Croatia, Ilica 244, 10000 Zagreb, Croatia

**Keywords:** pterygium, recurrence, gene expression

## Abstract

**Background/Objectives**: A pterygium is a fibrovascular ocular surface lesion whose recurrence after surgical excision remains a major clinical challenge, yet its molecular determinants of recurrence remain poorly understood. This study investigated transcriptomic profiles of primary pterygium tissue collected at the time of surgery to identify molecular signatures associated with subsequent recurrence. **Methods**: Eighty-five patients undergoing primary pterygium surgery were enrolled and followed for a mean of 7.32 years. RNA sequencing was performed on primary pterygium tissues collected at the time of surgery. Samples from four pterygia that developed recurrence and four non-recurrent pterygia were studied, with differential expression analyzed using DESeq2 and pathway enrichment via Reactome and GSEA. **Results**: Recurrence occurred in 5.8% of patients (mean time to recurrence was 8.87 months), exclusively in higher-grade (T2–T3) lesions. Transcriptomic analysis identified 1374 differentially expressed genes (1020 upregulated, 354 downregulated), showing separation between pterygia that developed recurrence from non-recurrent samples. Upregulated genes, including ECM1, HOXB5, MARCO, SHISAL1, CWH43, and PSORS1C1, reflected extracellular matrix remodeling and innate immune pathways, whereas downregulated genes, such as LCN10, ZBTB16, and GPR101, were linked to GPCR signaling and neuronal communication. Samples with the earliest recurrence displayed the most pronounced transcriptomic changes. **Conclusions**: These findings suggest that extracellular matrix remodeling and innate immunity activation, already present at initial surgery, are associated with subsequent pterygium recurrence and may help in the identification of candidate molecular biomarkers for recurrence risk stratification.

## 1. Introduction

Pterygia, wing-shaped non-carcinomatous lesions that grow on the ocular surface, are associated with eye symptoms caused by localized drying, inflammation, and visual disturbances. Pooled prevalence rises to 12%, especially in regions closer to the equator, suggesting a strong association between sun exposure and disease development [1]. UV exposure is a well-known risk factor, whereas additional environmental and host-related risk factors continue to be investigated. Ultraviolet (UV) radiation is thought to disrupt the barrier between the avascular cornea and the vascularized conjunctiva, while secondary insults, such as viral infection or further UV exposure, may subsequently promote cellular proliferation, hyperplasia, angiogenesis, tissue remodeling, and inflammation, ultimately leading to pterygium formation [2]. A recent study reveals insight into the association between pterygia and microbiota [3].

Although the exact pathogenesis of this fibrovascular lesion remains incompletely understood, additional factors such as dust exposure, chronic irritation, and persistent inflammation are believed to contribute to its development. Most of the current studies have based their research on possible new therapeutic approaches and surgical techniques, while the molecular mechanism of the formation and progression of pterygia is still poorly understood [4]. Based on current knowledge, it can be speculated that genetic changes occur at the level of conjunctival tissue under the influence of external factors, leading to a complex pathophysiological cascade of pterygium formation [5]. Monitoring changes in gene expression patterns, along with identifying upregulated and downregulated molecular pathways, may provide a clearer understanding of disease biology and serve as a potential predictor of therapeutic outcomes and relapse risk [5].

Currently, surgical excision remains the only effective treatment for pterygium; however, recurrence continues to represent a major clinical challenge, reaching up to 69.2% [6]. Patients undergoing bare sclera pterygium surgery had a greater five-year incidence of repeat pterygium excision than those undergoing surgery with a tissue graft (11.0% vs. 7.7%) [7]. The average global recurrence rates of conjunctival autograft (CAT), limbal conjunctival autograft (LCA), and amniotic membrane transplantation (AMT) are 7.61%, 5.50%, and 9.0%, respectively [8]. Although recurrence can still occur years later, most cases happen within the first 3–6 months after surgery [9]. The average time to recurrence with LCA was 8.8 months, while it was 4.8 months with CAT with sutureless fixation [10]. According to one meta-analysis, using fibrin glue instead of sutures may further lower recurrence rates, and its application is associated with lower recurrence rates, reduced operative time, and less postoperative discomfort [11].

This study investigated transcriptomic profiles of primary pterygium tissue collected at the time of surgery to identify molecular signatures associated with subsequent recurrence. We also examined the relationship between gene expression profiles, clinical pterygium subtype, and recurrence risk. We hypothesized that these analyses would facilitate the identification of factors associated with pterygium recurrence, thereby contributing to improved risk stratification and the development of strategies to reduce recurrence. In addition, this study provides updated data on long-term recurrence rates following conjunctival autografting. 

## 2. Materials and Methods

### 2.1. Study Participants and Procedures

We prospectively enrolled 85 patients undergoing primary pterygium surgery at the Department of Ophthalmology, University Hospital of Split, from September 2017 to January 2019. The inclusion criteria were a pterygium extending more than 4 mm onto the cornea and associated symptoms, such as visual impairment, chronic inflammation, or cosmetic concerns [12]. The exclusion criteria included the presence of any other ocular disease (e.g., glaucoma), regardless of the use of topical eye medications, as well as a history of previous ocular surgery. Written informed consent was obtained from all patients for sample collection and following analysis.

### 2.2. Data Collection

Pterygium severity was graded according to the classification system described by Donald Tan et al. (T1–T3), based on the visibility of the underlying episcleral blood vessels. T1 (atrophic) was defined as a thin, transparent pterygium with clearly visible episcleral vessels; T2 (intermediate) as a moderately thickened pterygium with partially obscured episcleral vessels; and T3 (fleshy) as a thick, opaque pterygium completely obscuring the underlying episcleral vessels [13]. To detect patients who were primarily operated on and later developed recurrence, electronic medical records were retrospectively reviewed and analyzed.

### 2.3. Surgical Technique

In this study, after excising the primary pterygium, conjunctival autograft was used and secured in place either with cauterization (using bipolar cautery) or with fibrin adhesive [12].

### 2.4. Tissue Samples

Surgical draping was performed in the standard manner. Samples of excised pterygium tissue overlying the cornea, containing both epithelial and stromal components, were collected at the beginning of the procedure to minimize tissue manipulation. The specimens were immediately placed in sterile Eppendorf tubes, snap-frozen in liquid nitrogen, and stored at −80 °C until mRNA isolation.

Among the five patients who developed recurrence, three underwent conjunctival autografting with graft fixation by cauterization and two with fibrin glue. The excluded sample belonged to the cauterization group; therefore, the four sequenced recurrent samples comprised two specimens from each fixation technique. The non-recurrent samples were matched accordingly, with two samples from each fixation group.

Importantly, all tissue specimens were collected at the beginning of the surgical procedure, before autograft harvesting and fixation. Therefore, the graft fixation technique could not have influenced the transcriptomic profile of the analyzed tissue specimens. However, due to the rather limited number of patients, the study is underpowered for detecting genes with a small effect size; thus, the findings are hypothesis-generating.

### 2.5. Histological and Staining Analysis

Tissue samples were fixed in 10% buffered formalin and routinely embedded in paraffin. Sections 4 μm thick were cut using a microtome (RM2125 RTS, Leica, Buffalo Grove, IL, USA) and subsequently stained with hematoxylin and eosin (Sigma-Aldrich, Darmstadt, Germany; Merck, Darmstadt, Germany) and Masson’s trichrome stain (BioGnost Ltd., Zagreb, Croatia).

### 2.6. RNA Extraction

Pterygium tissue specimens were collected during surgical excision and processed for nucleic acid isolation. Following collection, the tissue samples were snap-frozen and stored in liquid nitrogen until processing. The tissues were homogenized using the Minilys system (Bertin Technologies, Montigny-le-Bretonneux, France) using 1.4 mm zirconium oxide ceramic beads (Precellys, Bertin Technologies, Montigny-le-Bretonneux, France).

Total RNA was isolated with TRIzol reagent (Invitrogen, Carlsbad, CA, USA) according to the manufacturer’s protocol. Briefly, homogenized tissue was lysed in 0.25 mL TRIzol per sample, followed by phase separation using chloroform. After centrifugation at 12,000× *g* for 15 min at 4 °C, the aqueous phase containing RNA was transferred to a fresh RNase-free tube. RNA was precipitated with isopropanol, washed with 75% ethanol, air-dried, and resuspended in nuclease-free water (AccuGENE, Lonza, Basel, Switzerland).

RNA concentration was quantified using a NanoDrop spectrophotometer (ND-1000, Thermo Fisher Scientific, Waltham, MA, USA). Nucleic acid concentration was determined from absorbance at 260 nm, while purity was evaluated from the A260/A280 and A260/A230 ratios. Only four recurrent pterygium samples yielded a sufficient amount of RNA for library preparation and subsequent analysis.

### 2.7. RNA Sequencing

RNA sequencing was performed to characterize transcriptome-wide gene expression differences associated with subsequent pterygium recurrence. Total RNA isolated from pterygium specimens obtained during the initial surgical procedure was used for transcriptomic profiling. The final RNA-seq dataset comprised eight biological samples, including four samples from patients who subsequently developed recurrent pterygium and four samples from patients without recurrence during the defined follow-up period. The four non-recurrent samples were selected from the remaining non-recurrent patients by matching for Tan grade and graft fixation technique. Each tissue sample represented an independent biological replicate.

Library preparation and sequencing were performed by Novogene Europe (Novogene Co., Ltd., Cambridge, UK). Prior to library preparation, RNA integrity was evaluated using an Agilent 2100 Bioanalyzer (Agilent Technologies, Waldbronn, Germany). Samples were considered suitable for sequencing if they met the predefined quality and quantity criteria for RNA. Polyadenylated RNA was enriched from total RNA using oligo(dT)-conjugated magnetic beads. The enriched mRNA was fragmented to generate appropriately sized RNA fragments for library construction. First-strand complementary DNA (cDNA) was synthesized from fragmented RNA using random hexamer primers and reverse transcriptase. Second-strand cDNA synthesis was subsequently performed to generate double-stranded cDNA. A non-strand-specific (non-directional) RNA-seq library was prepared. Library construction included end repair, A-tailing, adaptor ligation, size selection, and PCR amplification. The resulting libraries were purified and assessed for concentration, fragment size distribution, and overall library quality using Qubit fluorometry, Bioanalyzer and qPCR.

Qualified libraries were pooled and sequenced on an Illumina NovaSeq platform (Illumina, Inc., San Diego, CA, USA) using 150 bp paired-end sequencing (PE150). Sequencing was targeted to a depth of approximately 40 million paired-end reads per sample. Raw sequencing reads in FASTQ format were subjected to quality control procedures before downstream analysis. Reads were filtered by removing those containing adapter contamination, reads with more than 10% uncertain nucleotides (N > 10%), and reads in which more than 50% of bases had a quality score below 5. Sequencing quality was evaluated using metrics including the percentages of bases with Phred quality scores of at least 20 and 30 (Q20 and Q30), GC content, and the number of retained clean reads. A total of 8 samples were analyzed in this sequencing for transcriptome analysis, resulting in 121.3 Gb of clean bases. Each sample produced over 12.17 of clean bases, with the Q20 base percentage greater than 99.24 and the Q30 base percentage greater than 96.71 for each sample. Each sample’s quality-controlled filtered reads were accurately aligned to the reference genome GRCh38/hg38 using HISAT2 version 2.2.1. The human reference genome was obtained from Ensembl. This provided the location information of the reads on the reference genome, with alignment rates for each sample ranging from 97.35 to 98.04. Gene-level read counts were generated using featureCounts version 2.0.6 on the Novogene pipeline component based on the corresponding genome annotation.

### 2.8. Differential Gene Expression and Statistical Analysis

Differential gene expression analysis was performed on protein-coding genes using DESeq2, package R version 1.42.0. DESeq2 models RNA-seq count data using a negative-binomial framework and estimates sample-specific size factors to account for differences in sequencing depth. The raw gene-count matrix was used as input, and samples were classified according to clinical outcome as recurrent (*n* = 4) or non-recurrent (*n* = 4). For each gene, DESeq2 was used to estimate the log2 fold change (log2FC) between recurrent and non-recurrent groups and the associated statistical significance. *p* values were corrected for multiple hypothesis testing using the Benjamini–Hochberg false discovery rate (FDR) procedure. Genes were considered differentially expressed when they met both an absolute log2 fold change ≥ 1 and an adjusted *p* value (FDR/q value) of <[0.1]. Accordingly, genes with log2FC ≥ 1 were classified as upregulated and genes with log2FC ≤ −1 as downregulated in recurrent relative to non-recurrent samples. A gene was considered to be expressed if the sum of its FPKM values across all samples was greater than 1.

Functional enrichment analyses were performed using NovoMagic, Novogene’s cloud-based bioinformatics analysis platform, which provides RNA-seq expression, differential expression, and functional analysis workflows. Specifically, clusterProfiler version 4.8.1 was used to perform GO and KEGG enrichment analysis. Gene sets with FDR < [0.1] were considered significantly enriched.

## 3. Results

### 3.1. Baseline Clinical Characteristics

A total of 87 subjects were initially assessed for eligibility in the study. Following the application of the inclusion and exclusion criteria, two subjects were excluded due to loss of follow-up. Therefore, 85 subjects were ultimately included in the final analysis. The baseline demographic and clinical characteristics of the 85 patients with pterygia are summarized in Table 1. The participants ranged in age from 24 to 83 years, with a mean age of 63.25 ± 12.71 years. Women accounted for 47.1% of the cohort (*n* = 40). Pterygium severity was further categorized into three grades. Grade 1 accounted for 21.2% of cases, grade 2 represented the largest proportion at 50.6%, and grade 3, indicating the most severe presentation, comprised 28.2% of cases (Table 1).

### 3.2. Incidence of Pterygium Recurrence and Tissue Staining of Recurrent and Non-Recurrent Pterygium Tissue Samples

**Figure 1:**
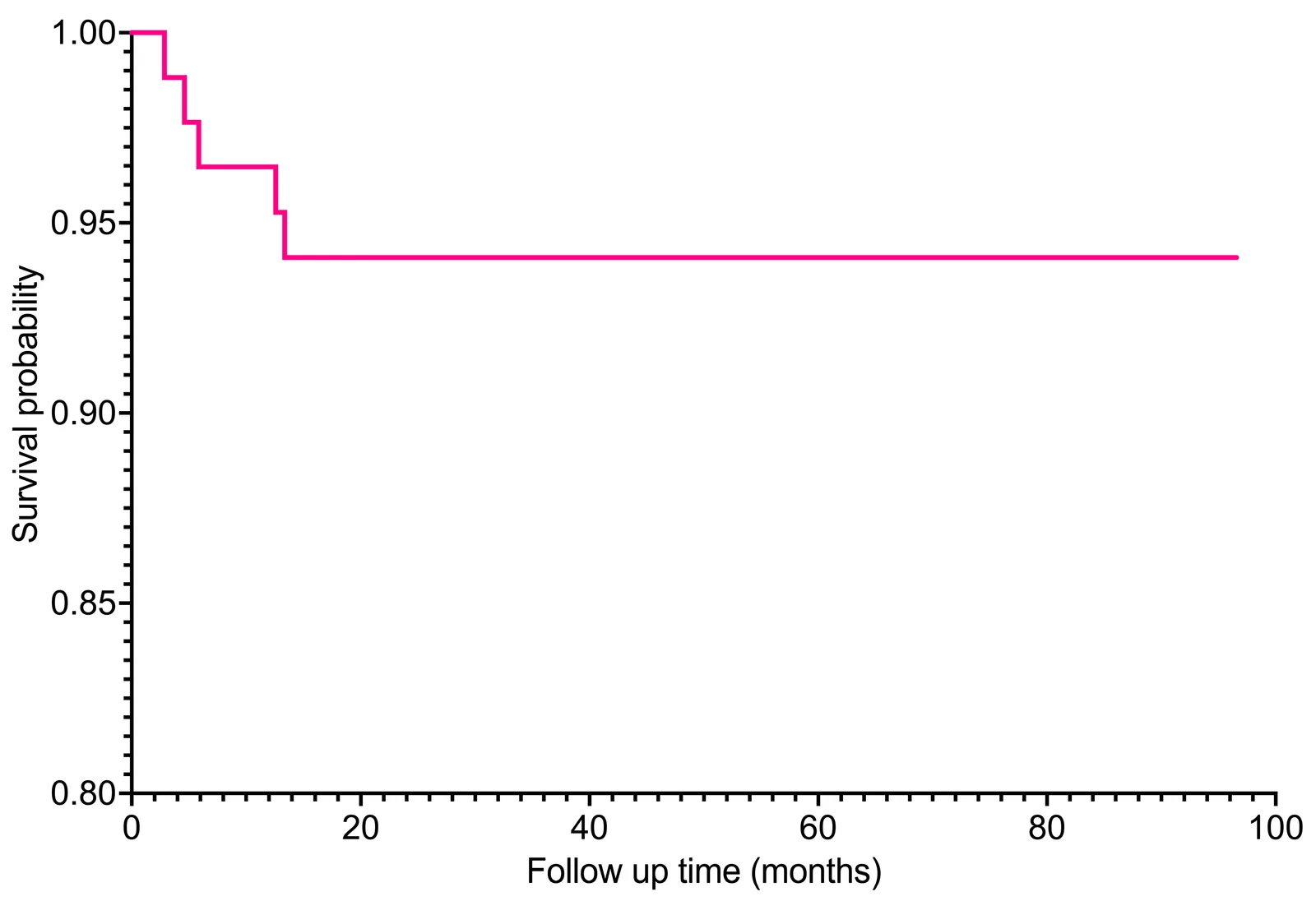
Kaplan–Meier Curve for pterygium recurrence.

All cases were classified as primary pterygium; however, recurrence occurred in 5.8% of patients (*n* = 5) during a mean follow-up period of 7.32 ± 1.69 years. The mean time to recurrence was 8.87 ± 5.15 months (0.74 ± 0.43 years), indicating that most recurrences developed within the first postoperative year, as represented in the Kaplan–Meier curve for pterygium recurrence (Figure 1).

A result of great clinical importance was that the pterygium samples that later developed recurrence were of grade 2, i.e.,T2, (four cases) and grade 3 i.e.,T3, (one case); none of the samples with the least severe clinical presentation, grade 1, i.e., T1 or thin pterygium, developed recurrence.

**Figure 2:**
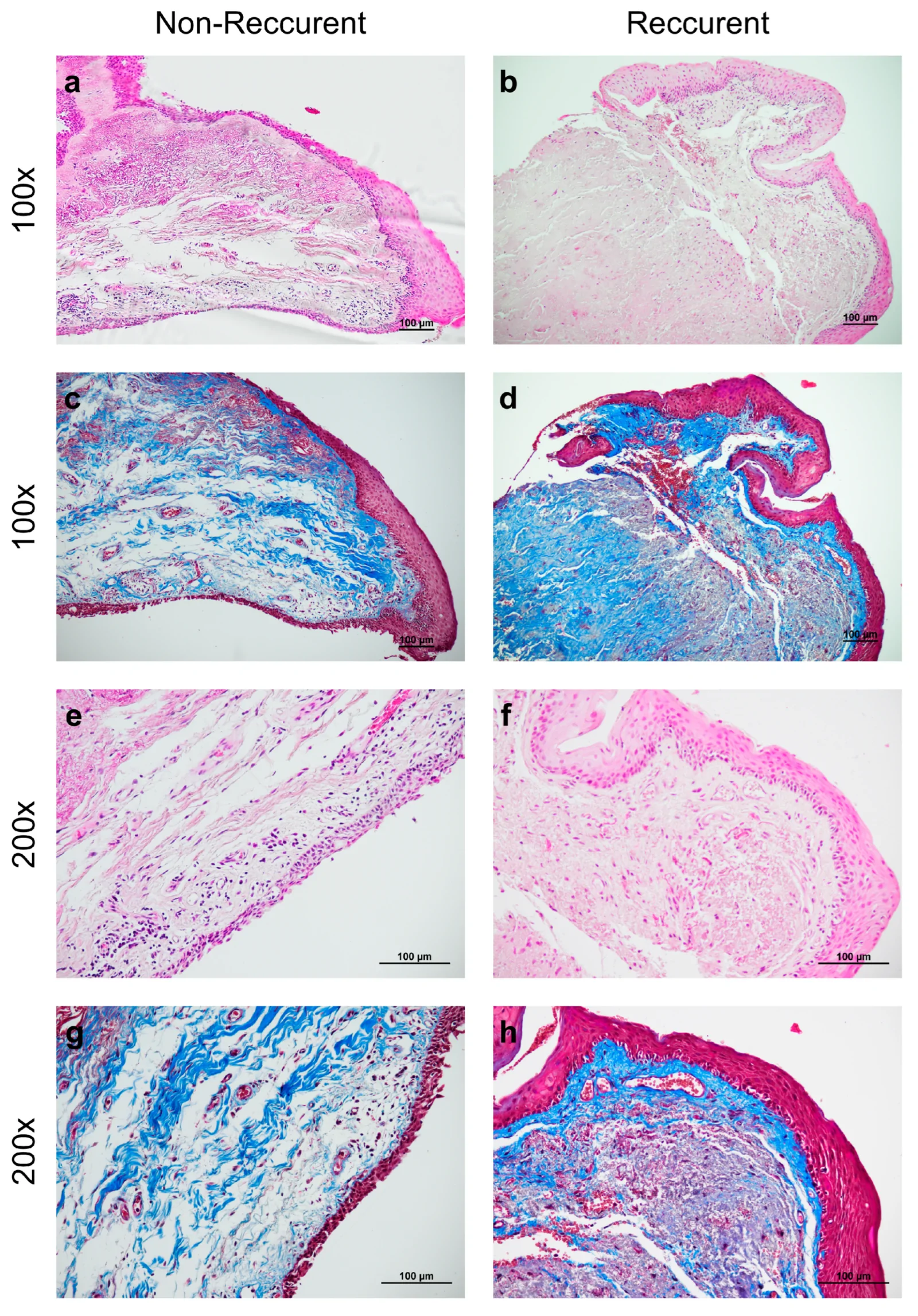
H&E (**a**,**b**,**e**,**f**) and Masson’s trichrome staining (**c**,**d**,**g**,**h**) of pterygium tissue. Blue-stained tissue elements in Masson’s staining represent collagen, i.e., connective tissue. Magnification is indicated on the figure panel.

Hematoxylin and eosin (H&E) staining of pterygium tissue demonstrated both epithelial and stromal alterations, including squamous metaplasia with loss of goblet cells in the epithelial layer, as well as fibrovascular connective tissue within the stroma composed of collagen, fibrillar structures, and blood vessels, accompanied by inflammatory infiltrates. Pterygium samples that developed recurrence exhibited increased cellularity in H&E staining and a more extensive fibrotic process on Masson’s trichrome staining. Representative images are shown in Figure 2.

### 3.3. Differential Gene Expression in Recurrent and Non-Recurrent Pterygium Samples

**Figure 3:**
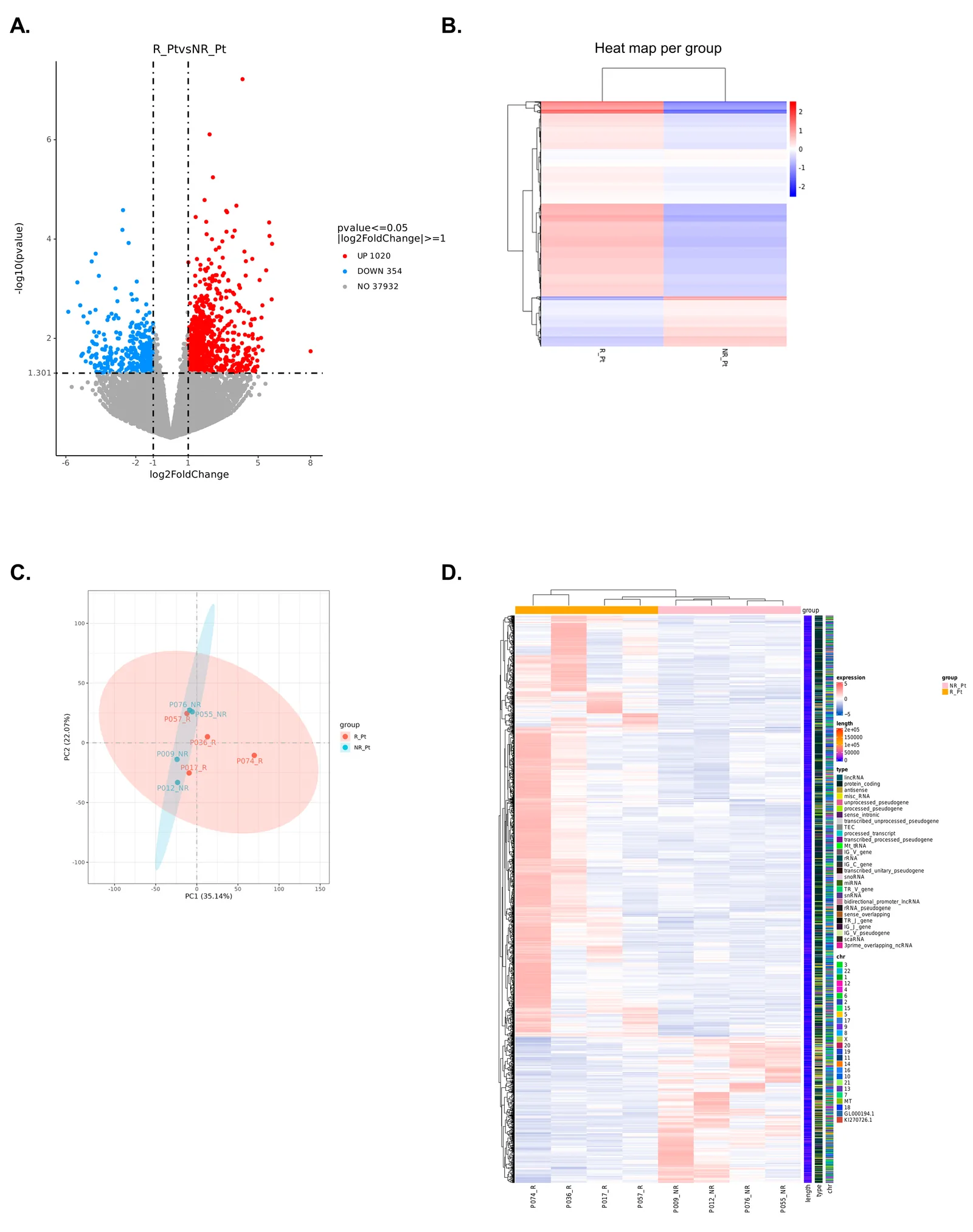
Gene expression profile of pterygium samples that did and did not recur. (**A**) Volcano plot showing genes differentially expressed in pterygium samples that subsequently recurred. (**B**) Heat map summarizing all samples in a group. (**C**) Principal component analysis of gene expression of recurring and non-recurring pterygium samples. (**D**) Heat map of individual samples from recurring and non-recurring pterygium samples.

Our transcriptomic analysis revealed significant differences in gene expression profiles between pterygium samples that subsequently developed recurrence and those that did not. Using a threshold of log2 fold change > 1 and a *p* value < 0.05, a total of 1374 differentially expressed genes were identified (Figure 3A). Among these, 1020 genes displayed increased (upregulated) expression, whereas 354 genes showed lower (downregulated) expression compared with genes from pterygium samples that did not recur. The volcano plot demonstrates substantial molecular differences between the two groups. A heat map summarizing the expression profiles showed a clear separation between the recurrent and non-recurrent pterygium samples (Figure 3B). To further assess overall transcriptomic variation, principal component analysis (PCA) was performed. The first two principal components (PC1 and PC2) explained 35.14% and 22.07% of the total variability among the samples, respectively (Figure 3C). Although the groups were largely separated, some overlap remained, indicating that the groups shared some transcriptomic similarities. Hierarchical clustering of gene expression profiles indicates differences in overall gene expression among individual pterygium samples (Figure 3D). Notably, the sample from the patient with the earliest recurrence (P074_R), which developed 3 months after surgery (Table 1), exhibited the most pronounced transcriptomic changes relative to non-recurrent samples. In contrast, sample P057_R, which developed recurrence after 13 months, displayed substantially fewer differentially expressed genes. Given the very small number of samples, this observation is exploratory and no relationship between the magnitude of transcriptomic change and time to recurrence can be inferred.

### 3.4. Upregulated Genes Supported by Gene Set Enrichment Analysis (GSEA) and Reactome Pathway Analysis in Pterygium Samples That Developed Recurrence

**Figure 4:**
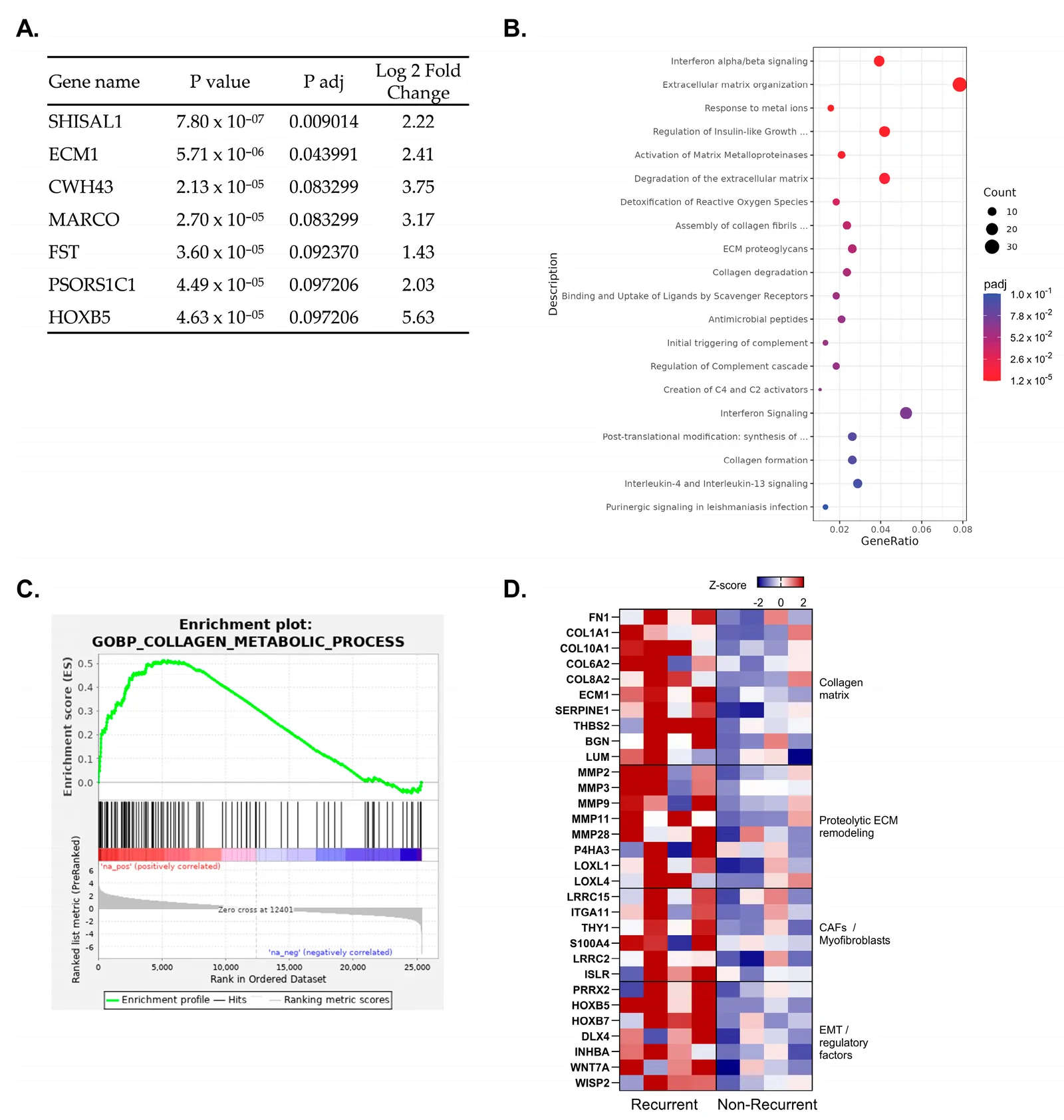
Upregulated genes and pathways associated with subsequent pterygium recurrence. (**A**) Top seven upregulated genes identified in pterygium samples that subsequently recurred. FDR < 0.1. (**B**) Reactome pathway enrichment analysis of upregulated genes. (**C**) Gene set enrichment analysis (GSEA) for the gene set related to collagen metabolic processes. GOBP—gene ontology biological process. (**D**) Heatmap showing the expression of genes in recurrent and non-recurrent pterygium samples, associated with a particular gene group. ECM, extracellular matrix; CAF, cancer-associated fibroblasts; EMT, epithelial–mesenchymal transition.

Among the pterygium samples that subsequently recurred, a distinct set of upregulated genes was identified. The most highly upregulated genes included ECM1, HOXB5, SHISAL1, CWH43, MARCO, and PSORS1C1 (Figure 4A).

Reactome pathway enrichment analysis demonstrated that the upregulated genes were predominantly associated with extracellular matrix (ECM) remodeling and innate immune responses (Figure 4B). Extracellular matrix-related pathways were the most prominently enriched, with seven of the top twenty Reactome pathways. Consistent with these findings, gene set enrichment analysis showed significant enrichment of collagen metabolic processes, indicating a positive association between enhanced collagen metabolism and subsequent pterygium recurrence (Figure 4C). In addition, a comparison of collagen matrix genes and genes involved in ECM remodeling displayed a clear distinction between pterygium samples that subsequently recurred and those that did not (Figure 4D). Additionally, genes involved in fibroblast activation and epithelial–mesenchymal transition are also upregulated in pterygium samples that subsequently recurred (Figure 4D). The remaining GO and KEGG enrichment pathway analyses are shown in Supplementary Figure S1.

Following ECM-related changes, enrichment analysis identified several pathways related to innate immune response-related pathways, although these exhibited lower scores and gene ratios (Figure 4B). These pathways were mainly driven by genes involved in complement activation and type I interferon response.

### 3.5. Downregulated Genes Supported by Reactome Pathway Analysis in Pterygium Samples That Developed Recurrence

**Figure 5:**
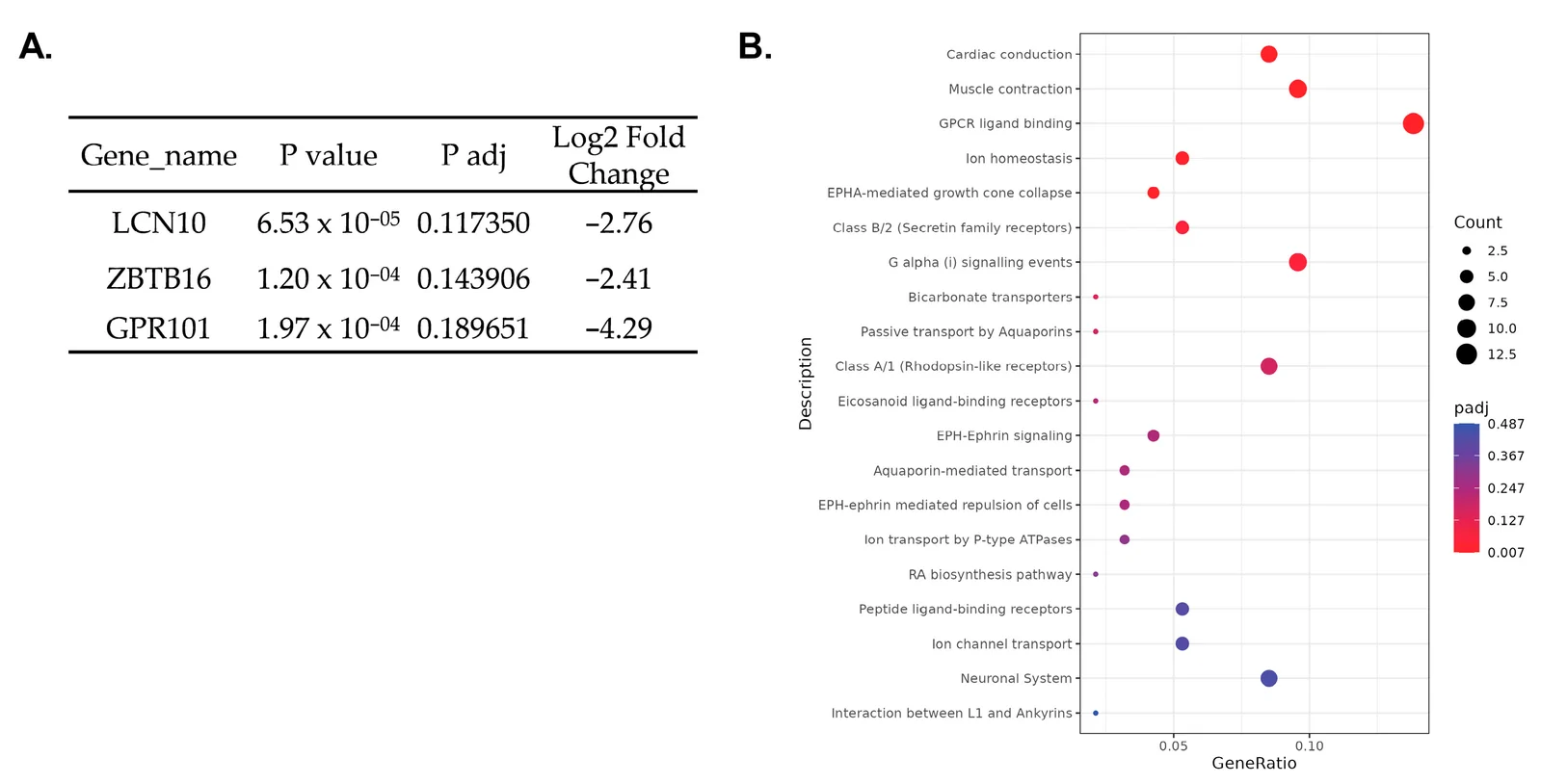
Downregulated genes and pathways associated with subsequent pterygium recurrence. (**A**) Top three downregulated genes identified in pterygium samples that subsequently recurred. FDR < 0.1. (**B**) Reactome pathway enrichment analysis of downregulated genes.

Among the pterygium samples that subsequently recurred, the most significantly downregulated genes were LCN10 (lipocalin 10); ZBTB16, a transcription factor; and GPR101, an orphan GPCR receptor. These genes represent biological functions related to carrier protein activity, transcriptional regulation, and orphan receptor-mediated signaling, respectively (Figure 5A).

The Reactome pathway enrichment analysis of downregulated genes revealed significant enrichment of pathways involved in G-protein-coupled receptor (GPCR) signaling, ion transport, neuronal communication, and muscle function (Figure 5B). Prominent pathways included GPCR ligand binding, G alpha (i) signaling events, ion channel transport, neuronal system, and EPH-ephrin signaling (Figure 5B).

## 4. Discussion

The main finding of this study is that the primary pterygium tissues that subsequently developed recurrence exhibited a distinct transcriptomic profile already present at the time of initial surgery. The most prominent molecular alterations involved extracellular matrix remodeling and innate immune pathways, suggesting that recurrence is associated with intrinsic biological characteristics of the primary lesion rather than postoperative wound healing alone. Clinically, the overall recurrence rate was 5.8%, consistent with published recurrence rates following conjunctival autografting [3]. Furthermore, all recurrent cases occurred in clinically more advanced pterygia (Tan grades T2 and T3), supporting previous observations that lesion severity is associated with an increased risk of recurrence [14]. Although histological analysis revealed only a trend toward higher cellularity in pterygia that later developed recurrence, distinct molecular differences were evident. Notably, recurrence occurred within the first postoperative year, and the sample with the earliest recurrence exhibited the most pronounced transcriptomic alterations. However, as recurrence occurred exclusively in clinically more advanced pterygia (Tan grades T2 and T3), earlier surgical excision at Tan grade T1 might reduce the risk of recurrence. This hypothesis, however, needs to be weighed against the fact that T1 lesions are frequently asymptomatic, and it should be tested prospectively before any change in surgical timing is recommended.

### 4.1. Extracellular Matrix Remodeling Activation Pathways

The upregulated gene expression profile suggests enhanced extracellular matrix (ECM) remodeling and the activation of immune-related pathways, both of which are consistent with pterygium pathogenesis [1]. The majority of the upregulated genes are associated with ECM organization and tissue remodeling, as evidenced by other studies. Notably, ECM1 (extracellular matrix protein 1) encodes a secreted glycoprotein that contributes to matrix organization, angiogenesis, and tissue homeostasis, in part through the inhibition of MMP9 [15]. CWH43 (cell wall biogenesis 43 C-terminal homolog) encodes a membrane-associated protein involved in glycosylphosphatidylinositol (GPI) anchor biosynthesis and membrane protein processing, processes that support tissue remodeling [16]. FST (follistatin), a secreted antagonist of transforming growth factor-β (TGF-β) family ligands, including activins, myostatin, and bone morphogenetic proteins (BMPs), regulates tissue growth, muscle development, and cellular differentiation [17]. Although primarily recognized as a developmental transcription factor, HOXB5 (homeobox protein Hox-B5) may also contribute to ECM remodeling through its roles in embryonic patterning, organogenesis, and skeletal morphogenesis [18].

### 4.2. Innate Immune Activation Pathways

In addition to ECM-associated genes, several upregulated genes are involved in immune regulation, another hallmark of pterygium pathogenesis. MARCO (macrophage receptor with collagenous structure) encodes a scavenger receptor expressed by macrophages that mediates pathogen recognition and clearance, whereas PSORS1C1 (psoriasis susceptibility 1 candidate 1) encodes a nuclear protein that has been genetically linked to the autoimmune disease psoriasis [19,20]. However, gene SHISAL (shisa like1) is poorly characterized with unknown function and could not be assigned a role in pterygium recurrence. Findings obtained from previous studies paralleled our data supporting the involvement of both aberrant ECM remodeling and immune activation in the molecular mechanisms underlying pterygium development and its recurrence.

Transcriptomic findings further suggest the coordinated downregulation of several genes, including LCN10, ZBTB16, and GPR101. Lipocalin 10 (LCN10) is involved in macrophage activation, response to lipopolysaccharide, stress response, and type II interferon response, further supporting the inflammatory changes that contribute to recurrent pterygium development [21]. Two additional downregulated genes, ZBTB16 (zinc finger and BTB domain-containing protein 16) and GPR101 (G protein-coupled receptor 101), are less well characterized and represent a transcriptional repressor and a signaling orphan receptor without obvious links to pterygium recurrence [22,23].

Since the up- or downregulation of genes was present at the time of initial surgery in pterygium-affected tissues, and only in pterygium cases that later developed recurrence, these changes could possibly be used as a biomarker for pterygium recurrence. However, our study was conducted at a single center, which may limit the generalizability of the results due to potential population- and institution-specific factors. Furthermore, the transcriptomic analyses were based on a relatively small number of samples and a single type of analysis, which may reduce statistical power and the ability to detect subtle but biologically relevant changes. Although transcriptomic analyses were performed on a limited number of recurrent samples because recurrence itself was uncommon, the consistency across principal component analysis, hierarchical clustering, Reactome pathway enrichment, and gene set enrichment analysis supports the fact that the observed transcriptomic signature reflects underlying biological differences rather than random variation. Nevertheless, these findings should be regarded as hypothesis-generating until validated in larger, independent cohorts.

## 5. Conclusions

Our findings demonstrate that primary pterygium tissues that subsequently recur exhibit distinct gene expression profiles at the time of initial surgery, characterized predominantly by extracellular matrix remodeling and innate immune activation. These transcriptomic signatures may represent candidate biomarkers for recurrence risk stratification and provide a foundation for future studies aimed at developing targeted strategies to reduce pterygium recurrence.

## Figures and Tables

**Table 1:** Baseline characteristics of subjects enrolled in study.

Parameter	Subjects (*n* = 85)	Recurrence (*n* = 5)
Age (year)	63.25 ± 12.71	
Sex—*n* (%)		
Male	45 (52.9%)	
Female	40 (47.1%)	
Tan score—*n* (%)		
I	18 (21.2%)	0
II	43 (50.6%)	4 (80%)
III	24 (28.2%)	1 (20%)
Follow up time (years)	7.32 + 1.69	
Time of recurrence (years)		0.66 ± 0.4

## Data Availability

All data and materials are available upon request due to GDPR restrictions.

## Supplementary Materials

The following supporting information can be downloaded at: https://www.mdpi.com/article/10.3390/jcm15187257/s1, Figure S1. KEGG and GO pathway analysis.
