# Supplementary material for: Gene Expression Signatures Associated with Pterygium Recurrence
Source: J Clin Med. 2026 Sep 18;15(18):7257. doi: 10.3390/jcm15187257 (PMC13607067; doi:10.3390/jcm15187257)
Supplement: Supplementary file 1 [file jcm-15-07257-s001.zip › jcm-4508770-supplementary.pdf]

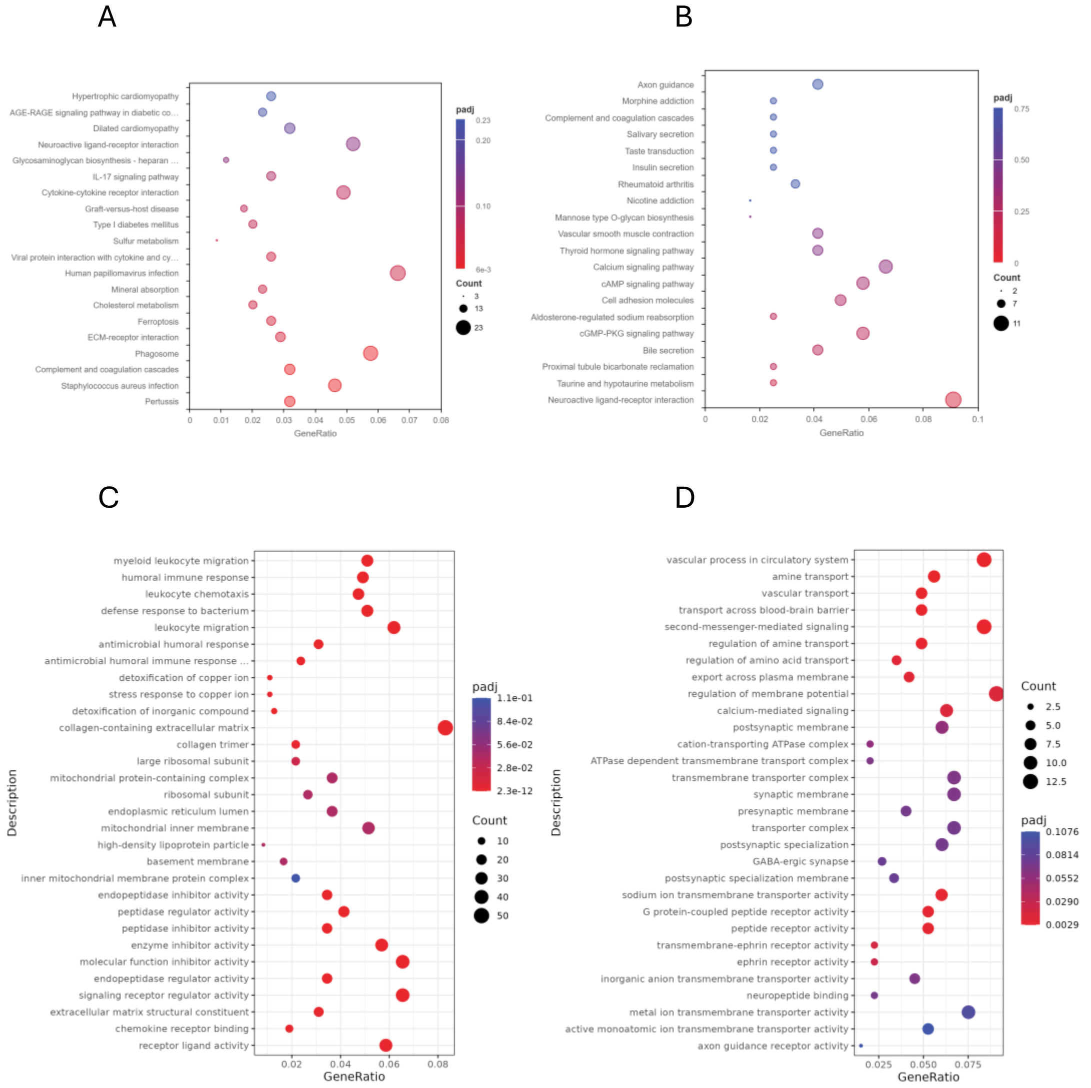

Supplementary Figure S1 KEGG and GO pathway analysis. **A**. Up-regulated pathways in recurrent pterygium tissue. **B**. Down-regulated pathways in recurrent pterygium tissue. **C.** Up-regualted GO pathways (Biological Process, Molecular Function, and Cellular Component) in recurrent pterygium tissue. **D.** Down-regualted GO pathways (Biological Process, Molecular Function, and Cellular Component) in recurrent pterygium tissue.
